# CT Attenuation Features of Individual Calcified Coronary Plaque: Differences among Asymptomatic, Stable Angina Pectoris, and Acute Coronary Syndrome Groups

**DOI:** 10.1371/journal.pone.0131254

**Published:** 2015-06-24

**Authors:** Yi-Luan Huang, Huey-Shyan Lin, Carol C. Wu, Fu-Zong Wu, Chinson Yeh, Kuan-Ran Chiou, Guang-Yuan Mar, Ming-Ting Wu

**Affiliations:** 1 Department of Radiology, Kaohsiung Veterans General Hospital, Kaohsiung, Taiwan; 2 Faculty of Medicine, School of Medicine, National Yang Ming University, Taipei, Taiwan; 3 School of Nursing, Fooyin University, Kaohsiung, Taiwan; 4 Department of Diagnostic Imaging, University of Texas MD Anderson Cancer Center, Houston, Texas, United States of America; 5 Department of Mechanical & Electro-Mechanical Engineering, National Sun Yat-Sen University, Kaohsiung, Taiwan; 6 Division of Cardiology, Department of Medicine, Kaohsiung Veterans General Hospital, Kaohsiung, Taiwan; 7 Institute of Clinical Medicine, National Yang Ming University, Taipei, Taiwan; Brigham and Women's Hospital, Harvard Medical School, UNITED STATES

## Abstract

**Background:**

Coronary artery calcium (CAC) assessed by non-contrast cardiac CT has been shown to be an independent factor from the Framingham risk factors in predicting cardiovascular events. However, many patients with acute coronary syndrome (ACS) have low CAC score. A recent study that re-analyzed the previous CAC CT scan of MESA cohort showed that in subjects with global lower density, CAC was associated with higher risk of ACS. We aimed to further evaluate the characteristics of CAC attenuation features in ACS subjects, in comparison to asymptomatic and stable angina pectoris (SAP) groups.

**Methods:**

In a period of 18 months, 524 consecutive subjects received standard CAC CT scans in our department; 278 of 524 subjects with presence of CAC (225 men, age = 60.6±9.5 years; ACS = 41, SAP = 78, asymptomatic = 159) were enrolled. Agatston score, number of plaques (N_P_) per subject and mean (H_MEAN_) and standard deviation (H_SD_) of attenuation of each calcified plaque were measured. Three regression models to distinguish the groups were built: model 1, conventional risk factors only; model 2, Agatston score plus model 1; model 3, plaque attenuation features plus model 2.

**Results:**

Agatston score in ACS group (median = 112.9) was higher than in the asymptomatic group (median = 54.4, P = 0.028) and similar to the SAP group (median = 237.8, P = 0.428). Calcified plaques in the ACS group showed lower (H_MEAN_ = 180.5) and more homogenous (H_SD_ = 31.2) attenuation than those of the asymptomatic group (H_MEAN_ = 205.9, P = 0.002; H_SD_ = 52.4, P = 0.006) and the SAP group (H_MEAN_ = 204.1, P = 0.016; H_SD_ = 54.4, P = 0.011). Model 3 significantly improved the distinction between ACS and asymptomatic groups (area under curve [AUC] = 0.93) as compared to model 2 (AUC = 0.83, P = 0.003) and model 1 (AUC = 0.79, P = 0.001).

**Conclusions:**

Calcified plaques in the ACS group were characteristically of low and homogenous CT attenuation. With validation in a large cohort, analysis of CT attenuation features may improve risk stratification of ACS using CAC CT scan.

## Introduction

The extent of coronary artery calcium (CAC) is thought to in part reflect coronary atherosclerotic plaque burden and has been used as a biomarker to predict the risk of cardiovascular events in recent decades [1–3]. Agatston score (AS) [4] derived from standard CAC CT scan, i.e. non-contrast ECG-gated cardiac CT scan, is currently the most widely used method to measure CAC [5]. AS has been shown to be an independent factor from the Framingham risk factors in predicting cardiovascular events in subjects with low or intermediate risk of coronary artery disease (CAD) [6–8] and in improving CAD risk stratification [9].

Although presence of CAC is a marker of atherosclerosis, its association with plaque stability seems to be less evident [10–12]. Global CAC score in many patients with acute coronary syndrome (ACS) was not high (AS <100 or zero) [13–16]. With advance of contrast-enhanced coronary CT angiography (CTA), non-calcified parts of vulnerable plaques can now be identified, with features such as adaptive remodeling, low attenuation core or napkin ring sign [15–17]; however, the features of calcified parts of vulnerable plaques, visible on non-contrast enhance scans, have not been well characterized except for the description of the so-called "spotty calcification" in mixed pattern plaque. Recently, a method to extract the density score of AS with re-analysis of the MESA cohort revealed that the CAC density (scored 1–4) is inversely and significantly correlated with risk of incident coronary event [18]. Moreover, a novel cardiac application of ^18^F-NaF PET has been reported to be able to identify the active calcification in the vulnerable plaques [19,20]; importantly, those with strong ^18^F-NaF uptake frequently have minimal CAC.

Given that nearly half of ACS patients have no previous angina symptoms [21], it would be of great clinical impact if standard CAC CT scan could be used to identify the features of calcified plaques of vulnerable subjects from the asymptomatic population in whom CAC CT scan is primarily indicated. In this study, we aimed to: 1) develop a dedicated tool to evaluate the attenuation features of individual calcified plaque, as individual plaque analysis may be complementary to global CAC score in assessment of coronary event risk [22]; 2) characterize the CT features of calcified plaques in asymptomatic subjects, patients with stable angina pectoris (SAP) and ACS prospectively enrolled during a fixed period; and 3) add the plaque features to traditional factors and AS to build a new model for risk stratification of coronary event.

## Materials and Methods

### Study populations

The Radiological Information System of the hospital was queried for cardiac CT exams performed from March 2012 to August 2013. Patients who suffered from ACS with no prior percutaneous coronary intervention or symptomatic patients with suspected or known CAD were reviewed (n = 159). The exclusion criteria are 1) absence of CAC on non-contrast cardiac CT scan (n = 16); 2) previous history of coronary bypass surgery or stent (n = 20); or 3) poor CT image quality due to motion artifacts (n = 4). As a result, 119 subjects, comprising 41subjects (35 men) in the ACS group and 78 subjects (69 men) in the SAP group were enrolled. During the same period, 345 asymptomatic self-referred subjects (258 men) underwent cardiac CT for health evaluation; among them, 159 subjects found to have CAC were enrolled in the asymptomatic group. Therefore, 278 subjects were enrolled (Table 1). This study was approved by the Institutional Review Board of Kaohsiung Veterans General Hospital. The Institutional Review Board waived the need for written informed consent from the participants.

**Table 1:** Clinical Characteristics and Distribution of Agatston Scores of the Three Subject Groups.

	Asymptomatic		SAP		ACS		
Clinical Characteristic	n = 159		n = 78		n = 41		p value*
Age, years (SD)	59.6	(8.6)	61.6	(10.7)	62.8	(10.2)	0.151
Male, n (%)	121	(76)	69	(88)	35	(85)	0.056
Body mass index, kg/m^2^ (SD)	25.2	(2.7)	25.7	(3.4)	25	(4.2)	0.319
Diabetes mellitus, n (%)	25	(16)	20	(26)	18	(44)	0.001
Hypertension, n (%)	72	(42)	42	(54)	30	(73)	0.007
Hypercholesterolemia, n (%)	81	(51)	36	(46)	17	(41)	0.390
Low HDL-C, n (%)	71	(45)	39	(46)	25	(58)	0.281
Smoker, n (%)	39	(25)	37	(47)	26	(63)	<0.001
Former	21	(13)	8	(10)	5	(12)	0.002
Current	18	(11)	29	(37)	21	(51)	
Rank of AS, n (%)							<0.001
1–10	30	(18.9)	7	(9.0)	3	(7.3)	
11–100	78	(49.1)	14	(17.9)	17	(41.5)	
101–200	25	(15.7)	16	(20.5)	5	(12.2)	
201–400	12	(7.5)	14	(17.9)	3	(7.3)	
> 400	14	(8.8)	27	(34.6)	13	(31.7)	

*by Chi-square test or ANOVA

ACS: acute coronary syndrome; AS: Agatston score; CAD: coronary artery disease; HDL-C = high-density lipoprotein cholesterol; SAP = stable angina pectoris

The diagnoses of ACS and SAP were made by cardiologists with 15 and 20 years of experience [K.R.C, G.Y.M.], according to the guidelines of the European Society of Cardiology and the American College of Cardiology/American Heart Association on the basis of clinical presentation, ECG finding, serum biomarkers, and coronary CT angiography [23,24]. Conventional risk factors, including sex, age, body mass index, diabetes mellitus, hypertension, dyslipidemia, and history of smoking were acquired from questionnaires, referral sheets, laboratory data and medical records, and determined according to published criteria [25].The history of smoking included current smoker (defined as persons who smoked regularly during the previous 12 months) and ex-smokers. Dyslipidemia included hypercholesterolemia (>200 mg/dL) or low high-density lipoprotein cholesterol (HDL-C<40 mg/dL for men and < 50 mg/dl for women).

### CT data acquisition

Beta-blocking medication (Metoprolol 50–200 mg, Selokeen, AstraZeneca, London, UK) was administered orally before the cardiac CT if a subject’s heart rate was greater than 65 beats per minute, in the absence of known contraindications. All scans were performed on a 64-slice MSCT scanner (Aquilion 64, Toshiba Medical Systems Corporation, Otawara, Japan). The cardiac CT protocol included a non-enhanced scan for CAC and a contrast-enhanced coronary CT angiography. The scanning parameters for quantification of CAC were as follows: prospective ECG-triggered sequential mode, tube voltage 120 kV, tube current 250–300 mA, gantry rotation time 0.4 sec., slice collimation 3.0 mm, and 180-mm field of view. The images were automatically reconstructed at 75% of the R–R interval using a 512 x 512 matrix, medium soft-tissue kernel, and 3.0-mm slice thickness with an increment of 3.0 mm.

### Data evaluation

Offline analyses of CT datasets were performed on an institutionally developed program. Segmentation of calcified plaque was based on a semi-automated algorithm called regional growing method[26], modified and customized by C.W.Y [27] for efficient assignment of each calcified plaque. The entire coronary arterial tree was inspected for the presence of calcified plaques. The number of calcified plaques (N_P_) and their location in the four major coronary arteries were recorded. A calcified plaque was defined as an area of 3 connected pixels with a CT attenuation> 130 HU applying 3D connectivity criteria [22]. In each calcified plaque, the acquired parameters included the length (L_P_), volume (V_P_), AS of individual plaque (AS_P_), mean of CT attenuation (H_MEAN_), standard deviation of CT attenuation (H_SD_), and coefficient of variation (CV) of CT attenuation (H_CV_) of individual plaque. H_CV_ was defined as the ratio of H_SD_ to H_MEAN_ of each calcified plaque. In this study, H_CV_ was multiplied by 100 for calculation of odds ratios. The AS was calculated according to the method introduced by Agatston et al [4]. For each subject, all AS_P_ were summed to AS and then converted into one of five ranks (1–10, 11–100, 101–200, 201–400, >400). The median of plaque parameters (i.e. AS_P_, V_P,_ L_P_, H_MEAN_, H_SD_, and H_CV_) of each subject was used for comparison.

### Validation study

To validate the accuracy of our program for AS calculation, we compared the AS calculated by our program to that of an FDA-approved program (VScore, Vitrea 2,V3.9.0.1, Vital Images, Minnetonka, MI, USA) for all cases by a thoracic radiologist with 3 years of experience (F.Z.W). We found high inter-method reliability (intraclass correlation coefficient ICC = 0.98). To evaluate the reproducibility of the plaque feature analysis by our program, a subset of 100 cases comprised of different AS ranks were randomly selected and re-analyzed by another thoracic radiologist with 10 years of experience (Y,L,H). We found that the inter-observer reliability was high for all parameters: AS_P_, N_P_,V_P_, L_P_, H_MEAN_ and H_SD_ (ICC = 0.94, 0.94, 0.95, 0.93, 0.95, 0.94 respectively).

### Statistical analysis

Categorical variables in different groups were expressed as frequencies and percentages, and were compared using the Chi-square test. Continuous variables in different groups were expressed as mean ± standard deviation (SD) or as median [25^th^, 75^th^ percentile] values, and were compared using the ANOVA for normal distribution and Kruskal-Wallis test for non-normal distribution and followed by the Steel-Dwass procedure method as post-hoc analysis. Conventional risk factors of CAD, AS ranks, and plaque parameters were used for multinomial logistic regression for associated predictors. We constructed three regression models for prediction. Model 1 included conventional risk factors only; Model 2 included conventional risk factors plus AS ranks; and Model 3 included factors used in model 2 plus plaque-feature parameters. The feature parameters were chosen through comparison of Akaike information criterion of different settings. All conventional risk factors were obliged for all three models, and AS and plaque-feature parameters were entered according to models. Furthermore, area under receiver-operating characteristic curve was used to compare the models (DeLong method). A p value less than 0.05 was considered to indicate a statistically significant difference for all tests. The analyses were performed using SPSS 19.0 for Windows (SPSS, Chicago, IL, USA) and R programming language (Team, R.D.C.2010, http://www.R-projecct.org) and MedCalc version 12.7.8 (MedCalc Software, Mariakerke, Belgium).

## Results

### Subject Characteristics

The clinical characteristics of the three groups, i.e. asymptomatic, SAP, and ACS, are summarized in Table 1. The asymptomatic group had 159 subjects and 773 plaques, the SAP group had 78 subjects and 801 plaques, and the ACS group had 41 subjects and 470 plaques. There were no significant differences in age (p = 0.151), body mass index (p = 0.319), and gender (p = 0.056) among the groups. Clinical risk factors of diabetes, history of smoking, and hypertension were significantly different among the three groups (all p < 0.05).

### Subject-based comparison of the three subject groups

The distribution of AS (Table 1) was significantly different among the three groups (p < 0.001). High AS (> 400) was equally prevalent in both SAP and ACS groups (p = 0.75), but significantly higher than that in the asymptomatic group (p < 0.01). The most prevalent AS in the ACS group was in the rank of 11–100 (41.5%), which was higher than that of the SAP group (p = 0.005) but not different from that of the asymptomatic group (p = 0.39).

Table 2 shows that there was no significant difference in AS between SAP and ACS groups, although the median value of SAP was double that of the ACS group (p = 0.43), while both groups had significantly higher AS than that of the asymptomatic group (p < 0.05). The total volume of calcified plaque per subject was not different between SAP and the ACS (p = 0.35) groups, and both were larger than that of the asymptomatic group (p < 0.001 and p = 0.001). The median numbers of calcified plaques per subject in SAP and ACS groups were similar (p = 0.82), but more than that of the asymptomatic group (p < 0.001 for both).

**Table 2:** Characteristics of Coronary Artery Calcification in the Three Subject Groups.

	Asymptomatic		SAP		ACS			Pairwise comparison		
patient number	n = 159		n = 78		n = 41			p value^†^		
plaque number	n = 773		n = 801		n = 470		p value*	#1	#2	#3
**per subject**										
AS	54.4	[13.5, 134.8]	237.8	[82.3, 543.6]	112.9	[17.1, 679.3]	<0.001	<0.001	0.028	0.428
Volume	49.0	[14.6, 119.6]	223.3	[88.3, 491.8]	113.2	[29.1, 622.0]	<0.001	<0.001	0.001	0.352
N_P_	3	[1, 6]	9	[4, 14]	8	[4, 20]	<0.001	<0.001	<0.001	0.822
**per plaque**										
AS_P_	13.4	[5.1, 20.5]	13.2	[4.8, 23.7]	6.0	[2.4, 19.6]	0.052	1	0.050	0.083
V_P_	13.9	[7.3, 20.1]	14.7	[7.8, 21.5]	9.1	[5.7, 18.8]	0.064	0.693	0.116	0.070
L_P_	3.8	[2.7, 5.0]	4.8	[3.6, 5.7]	3.6	[2.7, 5.4]	0.006	0.004	0.942	0.175
H_MEAN_	205.9	[181.3, 235.7]	204.1	[179, 224.5]	180.5	[166.9, 214.6]	0.003	0.930	0.002	0.016
H_SD_	52.4	[33.3, 77.1]	54.4	[34.4, 72.1]	31.2	[21.0, 62.1]	0.005	0.993	0.006	0.011
H_CV_	0.25	[0.18, 0.32]	0.26	[0.19, 0.33]	0.18	[0.13, 0.30]	0.012	0.850	0.018	0.016

Data are presented as median [25th, 75th percentile] values.

*by Kruskal-Wallis Test

^†^Post-hoc Steel-Dwass procedure test was performed for further pairwise comparison: #1 = Asymptomatic vs. SAP, #2 = Asymptomatic vs. ACS, and #3 = ACS vs. SAP.

H_CV_ means coefficient of variation of HU; N_P_: plaque number per subject; AS_P_: plaque-specific AS, L_P_: plaque-specific length (mm), V_P_: plaque-specific volume (mm^3^), other abbreviations as in Table 1.

### Plaque-based comparison of the three subject groups

AS_P_ and V_P_ of individual plaque were marginally different among the groups (p = 0.05, 0.06 respectively). The features of CT attenuation (i.e. H_MEAN_, H_SD_, and H_CV_) were all significantly lower in the ACS group than those in the SAP group and the asymptomatic group, while L_P_ was the only single plaque feature that was significantly different between asymptomatic and SAP groups (p = 0.004) (Table 2).

### Risk factors associated with the three subject groups

The comparison of risk factors associated with the three groups was analyzed by multinomial logistic regression model (Table 3).

**Table 3:** Multinomial Logistic Regression Analysis of Risk Factors Associated with the Three Subject Groups.

Predictors	SAP vs. Asymptomatic					ACS vs. Asymptomatic					ACS vs. SAP				
	p value	OR	95% CI			p value	OR	95% CI			p value	OR	95% CI		
**Conventional risk factors**															
Age	0.515	0.99	0.94	-	1.03	0.781	1.01	0.96	-	1.06	0.42	1.02	0.97	-	1.08
Sex (Male)	0.081	0.35	0.11	-	1.14	0.758	1.23	0.32	-	4.69	0.109	3.54	0.75	-	16.61
Body mass index	0.634	1.03	0.91	-	1.16	0.362	0.93	0.8	-	1.08	0.207	0.91	0.78	-	1.06
Diabetes mellitus^a^	0.627	1.25	0.51	-	3.03	0.013	3.68	1.32	-	10.22	0.041	2.95	1.04	-	8.33
Hypertension^b^	0.28	1.52	0.71	-	3.23	0.021	3.29	1.2	-	9.02	0.145	2.17	0.77	-	6.12
Dyslipidemia^c^	0.076	0.46	0.2	-	1.09	0.176	0.49	0.17	-	1.38	0.911	1.06	0.36	-	3.11
Smoking^d^	0.013	2.75	1.24	-	6.1	0.001	5.69	2.05	-	15.85	0.166	2.07	0.74	-	5.8
**Rank of Agatston score** ^**e**^															
11–100	0.738	0.81	0.24	-	2.73	0.037	6.89	1.13	-	42.09	0.035	8.47	1.16	-	61.6
101–200	0.173	2.69	0.65	-	11.14	0.123	5.54	0.63	-	48.73	0.528	2.06	0.22	-	19.5
201–400	0.032	5.46	1.16	-	25.7	0.089	7.66	0.73	-	79.85	0.779	1.4	0.13	-	14.94
> 400	0.002	12.89	2.6	-	64.04	0.005	24.7	2.68	-	227.83	0.572	1.92	0.2	-	18.25
**Plaque features**															
AS_P_	0.001	0.91	0.86	-	0.96	0.05	0.93	0.87	-	1	0.523	1.03	0.95	-	1.11
L_P_	0.023	1.64	1.07	-	2.52	0.007	2.05	1.22	-	3.45	0.427	1.25	0.72	-	2.15
H_CV_ *	0.496	1.02	0.96	-	1.09	0.005	0.88	0.81	-	0.96	0.002	0.86	0.78	-	0.95

* H_CV_ was multiplied by 100 for calculation of odds ratio. Other abbreviations as in Tables 1 and 2. Reference group

^a.^ no diabetes mellitus

^b.^ no hypertension

^c.^ no dyslipidemia

^d.^ non-smoker

^e.^ rank of Agatston score group = 1–10

Compared to the asymptomatic group, the significant risk factors of the SAP included history of smoking, AS of 200–400 or >400, and plaque features of lower AS_P_ and larger L_P_.

Compared to the asymptomatic group, significant risk factors of ACS included diabetes, hypertension, history of smoking, and AS of 10–100 or >400. For plaque features, larger L_P_ and smaller H_CV_ were all significant risk factors.

Compared to the SAP group, the significant risk factors of ACS included diabetes, AS of 10–100, and smaller H_CV_ of plaques.

### Three regression models for distinguishing the three subject groups

Conventional risk factors, AS, and plaque features were used to build three predictive models for distinguishing the three groups. Model 1 utilized conventional risk factors only, Model 2 used convention risk factors and AS, while Model 3 incorporated plaque features, including AS_P_, L_P_, H_CV_, in addition to conventional risk factors and AS. Table 4 lists the comparison of area under ROC curve (AUC) of the three regression models. Model 3 provided the best result. The greatest distinction of two groups was found between the asymptomatic group and the ACS group (AUC = 0.93), followed by distinction between the asymptomatic group and the SAP group (AUC = 0.85) and distinction between the SAP group and the ACS group (AUC = 0.83). The distinction between the ACS group and the asymptomatic group significantly improved from AUC = 0.83 in model 2 to AUC = 0.93 in model 3 (p = 0.003), and from model 1 (AUC = 0.79) to model 3 (p = 0.001). The distinction between the SAP group and the ACS group also improved significantly from AUC = 0.67 in model 1 to AUC = 0.83 in model 3 (p = 0.014).

**Table 4:** Comparison of Area under Curve in Three Regression Models* for Inter-group Comparison of the three subject groups.

Group	Area Under Curve			Pairwise comparison p value		
	Model 1	Model 2	Model 3	1 vs. 2	1 vs. 3	2 vs.3
SAP vs. Asymptomatic	0.70	0.81	0.85	0.003	<0.001	0.013
ACS vs. Asymptomatic	0.79	0.83	0.93	0.207	0.001	0.003
ACS vs. SAP	0.67	0.77	0.83	0.078	0.014	0.115

* Model 1: Predictors include conventional risk factors, including sex, age, body mass index, diabetes mellitus, hypertension, dyslipidemia, and history of smoking.

Model 2: Predictors include conventional risk factors plus rank of AS.

Model 3: Predictors include those in model 2 plus plaque features: AS_P_, L_P_ and H_CV_

Abbreviations as in Tables 1 to 3.

## Discussion

This is the first study to systematically investigate the attenuation features of individual calcified plaque on standard CAC CT scan in three different coronary symptom groups. We found that the plaque attenuation in the ACS group tended to be lower (lower H_MEAN_) and more homogenous (smaller H_SD_) compared to that of the asymptomatic and SAP groups. On the multivariate regression analysis, attenuation feature (H_CV_) of individual calcified plaque was the strongest independent differentiating factor between the ACS and SAP groups, and between the ACS and asymptomatic groups. In addition, adding plaque features to conventional risk factors and global CAC score remarkably improved the differentiation of the ACS group from the asymptomatic group in ROC analysis. These findings have very important clinical implications, since nearly half of ACS patients have no previous history of angina [21]. It should be emphasized that we did not aim to identify the individual vulnerable plaque in this study. It is known that with contrast-enhanced CT angiograms, the features of adaptive remodeling, napkin ring sign and spotty calcification are indicatives of vulnerable plaque [15–17]; however, that was not the aim of this study. By analyzing the attenuation features of calcified plaques, we hoped to maximize the utility of standard CAC CT scan, to identify the characteristic features of CAC plaques in vulnerable subjects from the asymptomatic population.

Histopathological analyses have shown that CAC quantification is an excellent method of assessing atherosclerotic plaque presence at individual artery sites [1,12,28]. Moreover, the amount of calcium correlates with the overall magnitude of atherosclerotic plaque burden [1,3]. Atherosclerotic calcification is now regarded as an organized, regulated process similar to bone formation and might represent an attempt by the arterial wall to stabilize itself, thereby minimizing the risk of plaque rupture [3,11]. However, this process may lead to increased local stress at the interface between a calcified and non-calcified atherosclerotic section where plaque rupture often occurs [12,28]. It is believed that the vessel is rendered less vulnerable to rupture only when extensive calcification has occurred, whereas the early or intermediate stages of calcification may actually enhance plaque vulnerability [28].This concept is recently validated by the novel application of ^18^F-NaF to detect the active calcification in vulnerable plaques in vivo [19].When correlating PET and CT findings, it was found that many foci of strong F18-NaF uptake had minimal or zero CAC on CT scan, while densely calcified plaques frequently had no 18F-NaF uptake [20]. In other words, foci of active calcification usually have low density or microcalcification on CAC CT scan.

An important recent study based on retrospective analysis of AS from MESA cohort confirmed the concept that higher CAC density may be protective [18]. CAC density (score 1 to 4) is inversely and significantly associated with CAD risk. Hence, for subjects with the same CAC volume, those with CAC of lower density score have higher risks of CAD event [18]; however, this density score is limited by the specifications of AS, which involves multiplying the plaque area by 1, 2, 3, or 4 depending on the maximal attenuation within each plaque. It does not reflect the attenuation distribution of all pixels within each plaque. This is the first study to directly show that both H_MEAN_ and H_SD_ of calcified coronary plaques were significantly lower in the ACS group than in the asymptomatic and SAP groups.

The distribution of AS ranks was significantly different among the three groups (Table 1). The ACS group showed a double-peak pattern; the first peak was AS 11–100 (41.5%), followed by a second peak of AS > 400 (31.7%). Contrarily, the asymptomatic group showed a trend toward AS 11–100, and the SAP group toward AS > 400. Our finding supports the observation that AS distribution in ACS is a divergent combination of that of the asymptomatic and the SAP groups [13,29]

Because of the divergence of AS in ACS group, we found that there is a complementary role of subject-based and plaque-based CAC analysis. The subject-based CAC analysis, i.e., the global CAC score or the AS, was most distinguish between the asymptomatic and SAP groups; while the plaque-based analyses (AS_P_) showed more distinguish between the ACS and the asymptomatic groups (Table 2). This trend was also observed in the multivariate analysis (Table 3). Our findings support the notion that individual plaque analysis may be complementary to global CAC measurements in evaluating coronary event risk [22].

**Fig 1:**
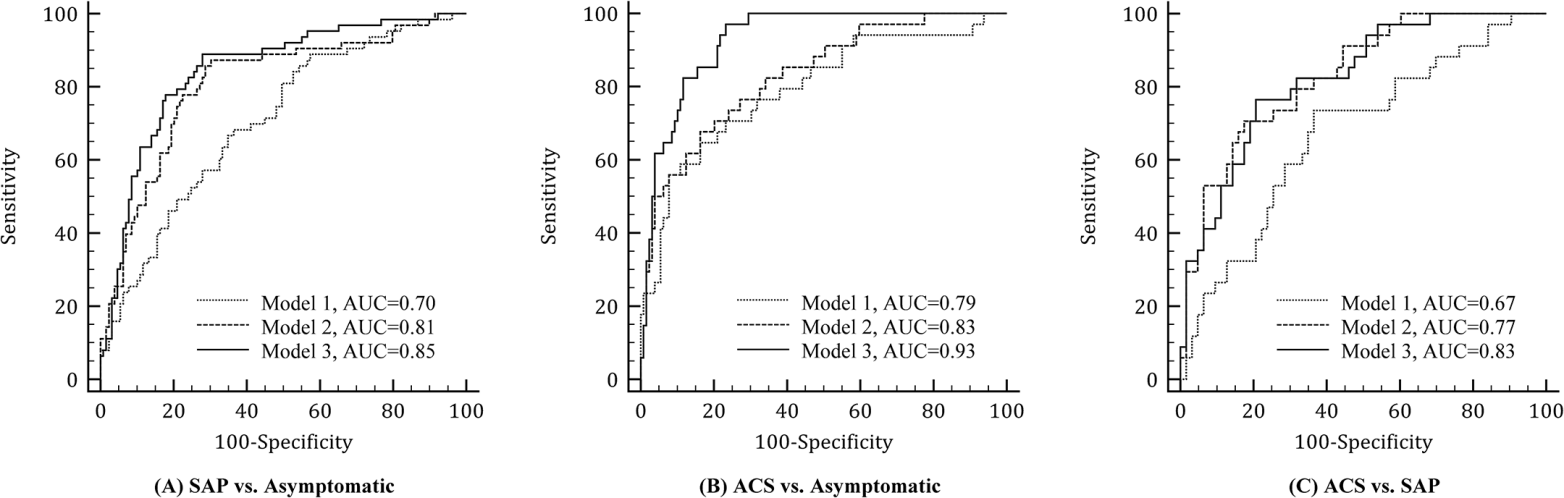
Receiver-operator characteristic (ROC) analysis for three regression models. ROC curves of three regression models to distinguish between (A) asymptomatic group and ACS group, (B) SAP group and ACS group and (C) asymptomatic group and SAP group. Model 1 included conventional risk factors only, Model 2 included conventional risk factors plus ranks of Agatston score, while Model 3 included those used in model 2 and plaque-feature parameters. ACS, acute coronary syndrome; SAP, stable angina pectoris.

Indeed, it is clear that the differentiation among the three groups was improved by adding plaque-based attenuation features in the model 3 of ROC analysis (Table 4, Fig 1). Of note, the differentiation between the ACS group and the asymptomatic group was significantly improved from AUC = 0.83 (model 2) to AUC = 0.93 (model 3) (p = 0.003). Given that approximately 40% to 60% of ACS occur as the first manifestation (unheralded events) of CAD [21], regression model 3 has important potential implications for primary prevention of ACS in the asymptomatic population in whom CAC CT scan is primarily indicated.

There are several limitations in the present study. First, the sample size is small and consists predominantly of Asian men. Second, the absence of follow-up of the asymptomatic group prevents correlation of the plaque features to eventual clinical outcomes; therefore, the plaque characteristics we observed in this cross-sectional study cannot be applied directly to predict the event. Finally, since the clinical significance of absence of CAC has already been demonstrated [30,31], the results of our prediction model 3 should be applied only to subjects with presence of CAC. A long-term cohort study with a large population of balanced genders is desirable to validate the prognostic impact of our proposed CAC attenuation features.

## Conclusion

We have developed a modified tool for attenuation feature analysis of individual calcified coronary plaques. We found that calcified plaques in ACS had significantly lower and more homogenous CT attenuation. With validation in future studies with large cohorts, the CT attenuation features based on standard CAC CT scan could potentially be utilized as a new biomarker to improve the risk stratification of CAD in asymptomatic subjects.
